# Case Report: Daratumumab in a Patient With Severe Refractory Anti-NMDA Receptor Encephalitis

**DOI:** 10.3389/fneur.2020.602102

**Published:** 2020-12-22

**Authors:** Dominica Ratuszny, Thomas Skripuletz, Florian Wegner, Matthias Groß, Christine Falk, Roland Jacobs, Heiner Ruschulte, Martin Stangel, Kurt-Wolfram Sühs

**Affiliations:** ^1^Department of Neurology, Hannover Medical School, Hanover, Germany; ^2^Clinic for Anaesthesiology and Intensive Care, Hannover Medical School, Hanover, Germany; ^3^Institute of Transplant Immunology, Hannover Medical School, Hanover, Germany; ^4^Department of Rheumatology and Clinical Immunology, Hannover Medical School, Hanover, Germany; ^5^Sana Clinic Hameln-Pyrmont, Hameln-Pyrmont, Germany

**Keywords:** anti-NMDA receptor encephalitis, autoimmune encephalitis, bortezomib, daratumumab, critical care

## Abstract

Anti-NMDA receptor encephalitis is the most common type of antibody mediated autoimmune encephalitis (AIE). Patients often develop neuropsychiatric symptoms and seizures, women are affected about four times more than men, and in about 50% the disease is associated with a neoplasia, especially teratomas of the ovary. We describe the case of a 20-year-old woman suffering from a severe therapy refractory course of anti-NMDA receptor encephalitis. Treatment included glucocorticoids, plasma exchange, intravenous immunoglobulins, rituximab, and bortezomib without clinical improvement. Due to a therapy refractive course 28 weeks after disease onset, the patient received 10 cycles of daratumumab. Therapy escalation was performed with the anti-CD38 monoclonal antibody daratumumab as off label treatment, based on the therapy of refractory myeloma and led to an improvement of her clinical status. She spent about 200 days on the intensive care unit, followed by several weeks on the intermediate care unit with close follow ups every 4–6 weeks afterward. During follow-up, the patient was able to resume everyday and self-care activities, reflected by the modified Rankin scale (mRS) and Barthel index. Because this disease is potentially life threatening and can lead to irreversible brain atrophy, development of further therapy strategies are of great importance. Our case describes a successful treatment for therapy refractory anti-NMDA receptor encephalitis using the anti-CD38 antibody daratumumab.

## Introduction

Anti-NMDA receptor encephalitis is an antibody-mediated autoimmune encephalitis associated with neuropsychiatric symptoms with a potentially life-threatening character. We report the case of a severe refractory course of anti-NMDA receptor encephalitis, with clinical improvement after the use of the anti-CD38 antibody daratumumab. The facultative paraneoplastic anti-NMDA receptor antibodies cause an autoimmune encephalitis (AIE), typically leading to psychiatric symptoms, seizures, involuntary movements, and autonomous dysregulation (1). Generally, first-line treatment consists of corticosteroids and plasma exchange or intravenous immunoglobulins (IVIG). Due to the antibody-mediated pathophysiology, rituximab is used as an early escalation treatment targeting CD20 positive cells (2). Refractory AIE therapies are derived from treatments of other autoimmune diseases, including cyclophosphamide and the proteasome inhibitor bortezomib used for multiple myeloma therapy (3). Daratumumab, an anti-CD38 monoclonal antibody that is approved for refractory myeloma, directly targets CD38 positive plasma cells and other lymphoid and myeloid cell populations (4).

## Case Report

In May 2017 a 20-year-old woman complained about headache and gastrointestinal symptoms. A few days later she developed fever, hallucinations, disturbed consciousness, and personality changes. On hospital admission about 1.5 weeks later, a cranial MRI depicted lesions in both mesial temporal lobes, suspicious for herpes encephalitis. Accordingly, i.v. acyclovir treatment was started. Additionally, she received prednisolone. For further treatment, she got transferred to a secondary care center in Germany about 2 weeks after symptom onset. Due to insufficient protective reflexes and seizures, orofacial, oculomotor, and extremity dyskniesia, she was intubated on the admission day and subsequently tracheotomized for long-time ventilator support. Tiapride, biperiden, and tetrabenazine had only limited effects on the choreoathetotic movements. Seizure therapy with levetiracetam was extended with topiramate and lacosamide. Repeated EEGs showed moderate slow activity with delta brushes.

**Figure 1:**
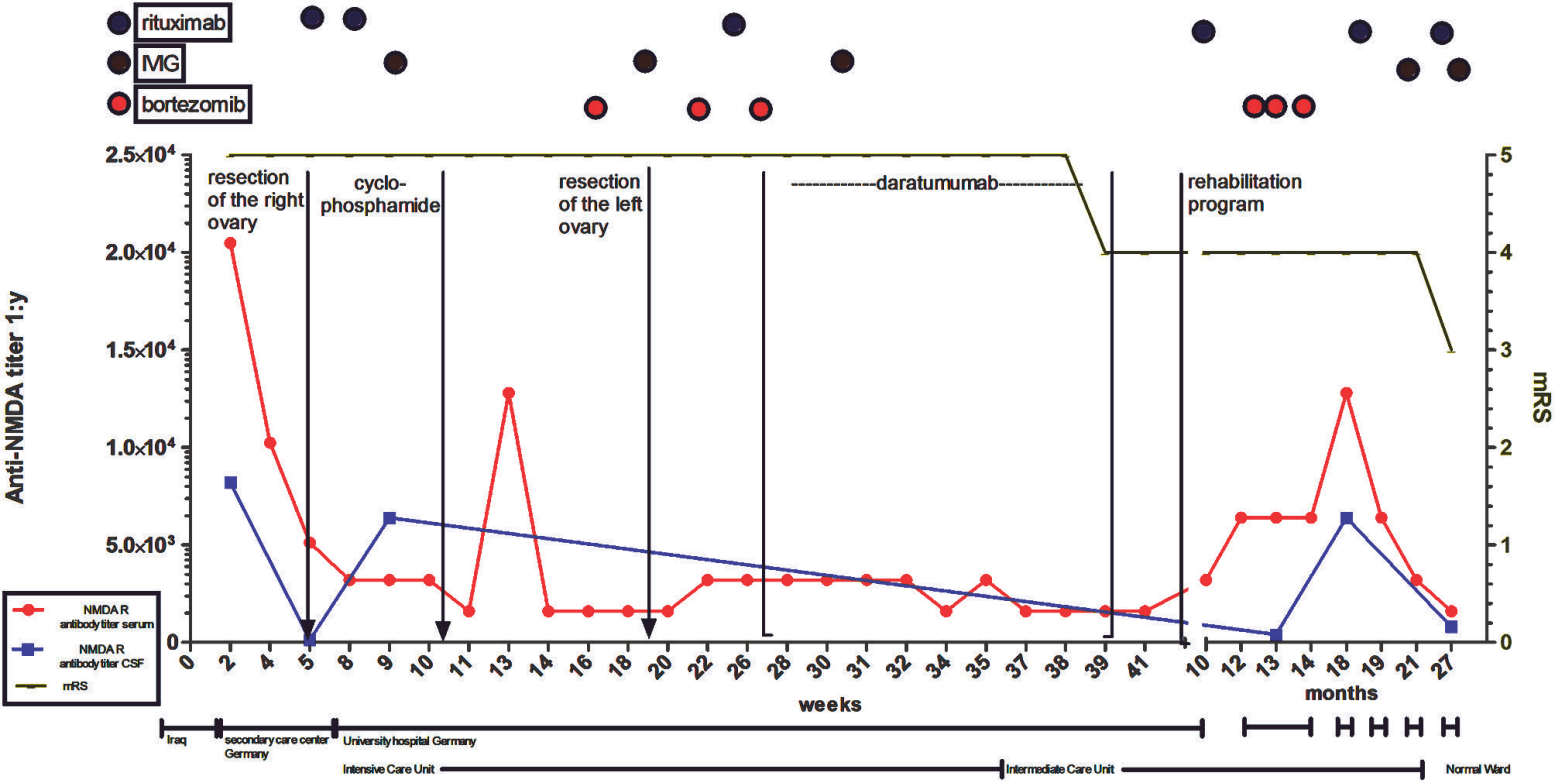
Illustration of treatment escalation in refractory course of anti-NMDA receptor encephalitis with special focus on antibody titer in CSF and serum as well as the clinical development over 27 months. mRS: modified Rankin scale (0, No symptoms; 1, No significant disability; 2, Slight disability, able to look after own affairs without assistance, but unable to carry out all previous activities; 3, Moderate disability. Requires some help, but able to walk unassisted; 4, Moderately severe disability. Unable to attend to own bodily needs without assistance, and unable to walk unassisted; 5, Severe disability. Requires constant nursing care and attention, bedridden, incontinent, 6 – Dead).

Lumbar puncture revealed lymphocytic pleocytosis (150 cells/μl). Positive oligoclonal bands and anti-NMDA-receptor antibodies were found in cerebrospinal fluid (CSF) and serum (Figure 1), as well as anti-AMPAR- and anti-mGLUR5-antibodies, both in serum and CSF, which allowed the diagnosis of autoimmune limbic encephalitis. The titer of anti-AMPA-R antibody in serum was <1:20. Anti-AMPA-R was only found in the first CSF sample taken in Germany, and the titer was low (1:4). mGLUR5 antibody titer in serum was 1:80. This antibody was found also in the first CSF taken in Germany (1:128) but during follow-up the titer decreased to 1:1.

Although, because multiple antibodies were detected, allowing the diagnosis of limbic encephalitis, the clinical stages were typical for an anti-NMDA receptor encephalitis (RE) (5). Viral polymerase chain reaction (PCR) including Herpes simplex virus (HSV) was negative. Radiological diagnostics showed pulmonary embolisms and a teratoma of the right ovary that was verified via biopsy and resected. After diagnosis of AIE, plasmapheresis combined with high-dose corticosteroids was started, followed by the first cycle of rituximab. Seven weeks after onset without any improvement of neurological status, she was transferred to our department [modified Rankin scale (mRS) 5, Barthel Index 0]. Again, plasmapheresis was performed, and corticosteroids were given, followed by the second course of rituximab. Immunomodulatory therapy was intensified with IVIG and cyclophosphamide. While anti-NMDA-receptor-antibody persisted in the blood and CSF, AMPAR and anti-mGLUR5-antibodies were undetectable in the serum and CSF. Every antibody detection in the serum or CSF in both hospitals in Germany was performed using cell-based assays.

**Figure 2:**
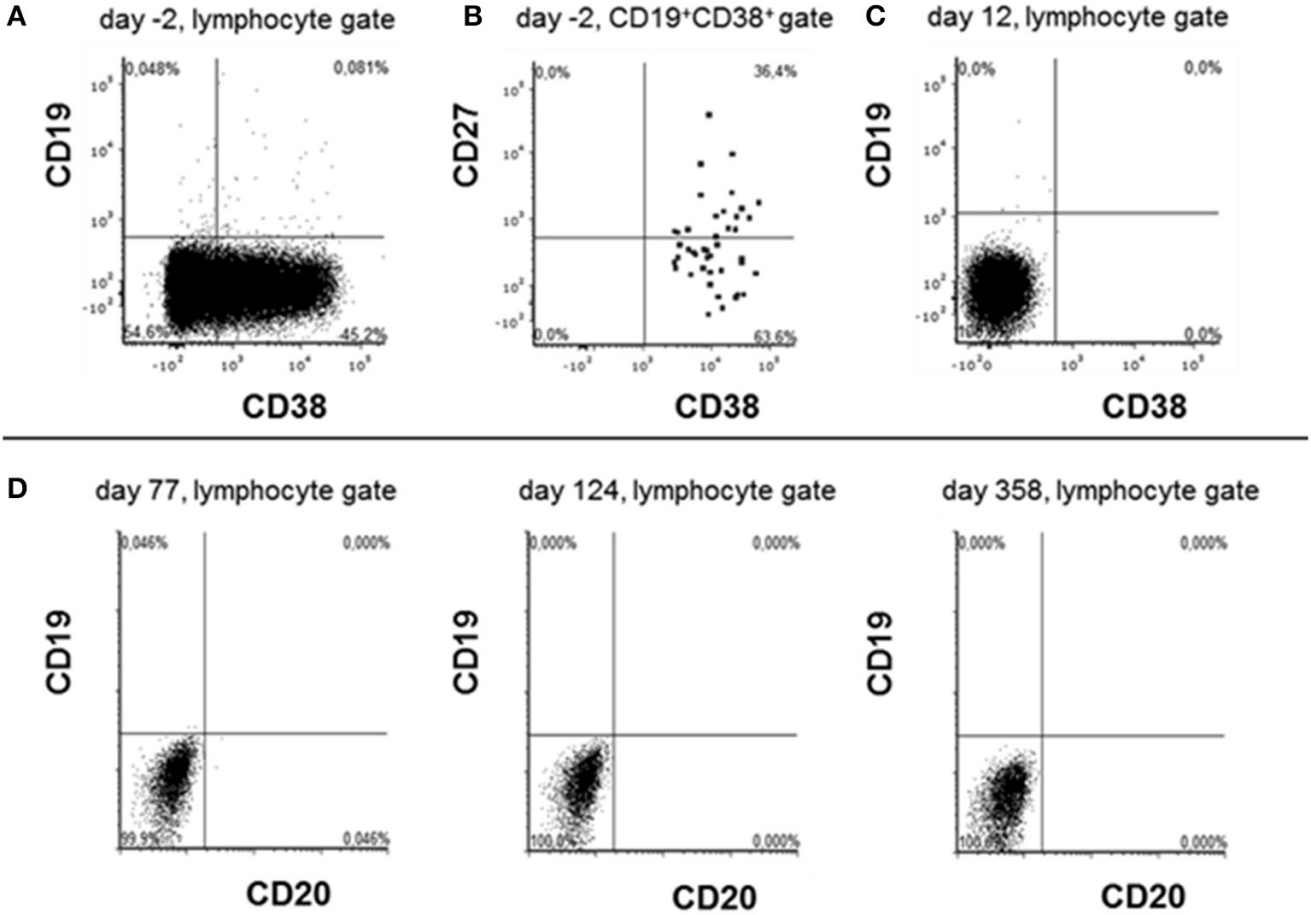
Phenotyping lymphocytes before and after daratumumab treatment. Peripheral blood lymphocytes were analyzed by flow cytometry 2 days before **(A,B)** and 12 days after daratumumab **(C)** has been administered. Due to prior rituximab application CD20/CD19high B cells were lacking. Cells were first gated on singlet lymphocytes according to their FSC vs. SSC properties and then analyzed in the gates indicated on the top of each dot plot. Dots stained positive in the CD19+CD38+ gate are shown emphasized for better visibility. **(D)** Depict data obtained during cytometric rituximab monitoring showing the absence of any CD19+ cells at the indicated time points after first daratumumab application.

Since there was no clinical improvement 16 weeks after onset, three cycles of bortezomib (1.3 mg/m^2^ on day 1, 4, 8, and 11 for each cycle) were administered. After recurrent urinary tract infections, the patient developed a septic shock and a cytomegalovirus (CMV)-associated pneumonia, forcing us to interrupt the bortezomib therapy. Extended imaging, including MRI of the thorax, abdomen, and pelvis, as well as PET-CTs with different tracers (FDG, CXCR4) did not show any type of neoplasia or abnormal lymphoid tissue. Following various publications, the left ovary was removed, yet no occult teratoma was found (6, 7). The clinical state remained unimproved; anti-NMDA receptor antibody titers showed a stable high level (1:1,600) (Figure 2), while a peripheral fluorescence-activated cell sorting (FACS) analysis rendered CD20 cell depletion after rituximab. For interalia based on the assumption that these antibodies were secreted by long-lived plasma cells or plasmablasts not affected by rituximab, we aimed to target this cell population. Approximately, 0.81% of all lymphocytes were CD19low and CD38 positive cells, 36.4% of which co-expressed CD27, fulfilling the marker criteria of plasma cells/plasmablasts in peripheral blood (Figures 2A,B), yielding 0.029% of all lymphocytes or 41/ml (normal range: 1–635/ml) (8). Based on the treatment of multiple myeloma, we applied the anti-CD38 antibody daratumumab in order to deplete these plasma cells. Thus, 16 mg daratumumab per kg bodyweight per cycle were given intravenously 28 weeks after disease onset, which corresponds to a total of 800 mg per cycle. According to the manufacturer's instructions, the first eight cycles were administered weekly, then the interval was extended to biweekly. For infusion therapy, daratumumab was dissolved in 1,000 ml NaCl 0.9 % and applied at 50 ml/h. Premedication consisted of prednisolone (100 mg/5 ml), clemastine (2 mg/5 ml), and paracetamol (1,000 mg/100 ml), which was administered 1 h before each cycle. She received a total of 10 intravenous cycles within 13 weeks. Flow cytometric monitoring of CD38 expression after daratumumab treatment is difficult. The daratumumab target epitope overlaps with the epitopes recognized by widely used monoclonal CD38 antibodies (e.g., HB7, HIT2) blocking the binding of the latter antibodies. The lack of detecting CD38+ cells, including all antibody-secreting cells such as plasma cells/plasmablasts, therefore indicates either the depletion or the saturated covering of the targeted cells unavoidably subjecting them soon to death (Figure 2C). In addition, the nearly comlette absence of CD19+ cells confirms at least a complete plasmablast depletion for nearly 1 year after starting daratumumab therapy (Figure 2D). Over the next 2 months, we observed reduced dyskinesia and an improved level of consciousness and a remission of care dependency and improvement of motor functions, allowing the patient to sit upright (Barthel index 5). The patient could be transferred to intermediate care 9 months after disease onset and 220 ICU days, followed by a rehabilitation program (Figure 1). Twelve months after onset, she received another three cycles of bortezomib due to the persistence of poor medical condition and rising anti-NMDA receptor antibody titers. Even so, the correlation of anti-NMDA-antibody titer and the patient's symptoms is poor as described by Gresa-Arribas et al. and reflected by this case (Figures 1, 2) (9). Rituximab (1,000 mg) was continued every 6 months after disease onset and IVIG (1–2 g/kgBW/d for 3 days) was given four times after initial administration. Eighteen months after disease onset, the patient was spontaneously breathing without dysphagia; hence, the tracheal cannula could be removed. Her state of consciousness fluctuated between awake and somnolent. She was able to say single words but without orientation, preferably sitting or lying in bed. Nine months after daratumumab treatment, she was awake and cooperative, orientated for her person, and location, able to have a basic conversation, and walking independently (Barthel index 80). On the last follow-up, 17 months after daratumumab, the patient was able to take care of herself and used a smartphone to write text messages, yet she was unable to take care for her legal matters.

Regarding the NEOS score, which in this case is four points, the likelihood of poor functional status is nearly 70% (10). After observing the patient's development over more than 2 years, we still see improvement with the ability for basic self-care at the moment. However, because the last cranial MRI showed global brain volume reduction, we apprehend persistent deficits of cognitive functions.

**Figure 3:**
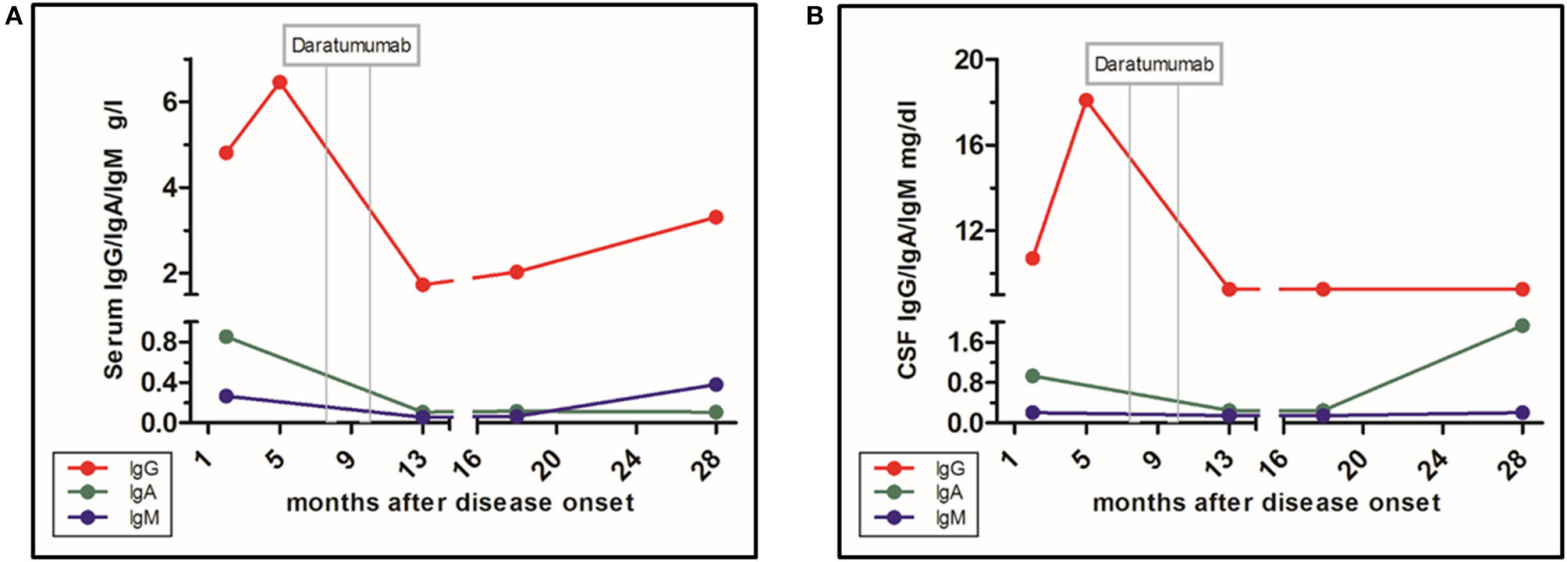
Level of immunoglobulin classes and influence of daratumumab. **(A)** Immunoglobulins in serum. **(B)** Immunoglobulins in CSF.

According to the guidelines, immunosuppression is essential for treatment of anti-NMDA receptor AIE (11). So far, randomized treatment trials are unavailable. We are aware of the fact that one single case cannot be taken as a guideline for further cases. However, in this case, targeting plasma cells, using the anti-CD38 antibody daratumumab analogous to refractory myeloma, has led to a positive turn in this refractory case. Of course the possibility of a spontaneous healing has to be considered, although patients with documented recovery without any treatment tend to have a milder course. Another limitation is that the sequential immunosuppressive treatment, including bortezomib, does not allow us to ascribe the clinical improvement to daratumumab alone (12, 13). Furthermore, the persisting anti-NMDAR titer, despite falling IgG levels, indicates a specific non-depleted plasma cell population possibly in the CNS in this case (Figure 3). One other case with a positive influence of daratumumab has been published, in a patient with a CASPR2-positive encephalitis, yet in that case, long-term follow-up was unavailable (14).

## Data Availability Statement

The raw data supporting the conclusions of this article will be made available by the authors, without undue reservation.

## Ethics Statement

Written informed consent was obtained from the individual(s) for the publication of any potentially identifiable images or data included in this article.

## Author Contributions

DR collected and analyzed the data and drafted the manuscript. TS, FW, MG, HR, and MS contributed in drafting the manuscript. RJ analyzed the data and contributed in drafting the manuscript. K-WS conceived the study, analyzed the data, and drafted the manuscript. All authors read and approved the final manuscript.

## Conflict of Interest

The authors declare that the research was conducted in the absence of any commercial or financial relationships that could be construed as a potential conflict of interest.
